# Efficacy of Digital Dance on Brain Imagery, Cognition, and Health: Randomized Controlled Trial

**DOI:** 10.2196/57694

**Published:** 2024-07-30

**Authors:** Heng-Hsin Tung, Chen-Yuan Kuo, Pei-Lin Lee, Chih-Wen Chang, Kun-Hsien Chou, Ching-Po Lin, Liang-Kung Chen

**Affiliations:** 1 Department of Nursing National Yang Ming Chiao Tung University Taipei Taiwan; 2 Tungs’ Taichung MetroHarbor Hospital Taichung Taiwan; 3 Center for Healthy Longevity and Aging Sciences National Yang Ming Chiao Tung University Taipei Taiwan; 4 Institute of Neuroscience National Yang Ming Chiao Tung University Taipei Taiwan; 5 Brain Research Center National Yang Ming Chiao Tung University Taipei Taiwan; 6 Department of Education and Research Taipei City Hospital Taipei Taiwan; 7 Center for Geriatrics and Gerontology Taipei Veterans General Hospital Taipei Taiwan; 8 Taipei Municipal Gan-Dau Hospital Taipei Taiwan

**Keywords:** digital, somatosensory dance, somatosensory game, cognitive performance, physical function, resilience, demoralization, quality of life, dance, dancer, dancing, movement, sport, sports, cognitive, cognition, brain, neuroscience, image, imagery, imaging, RCT, randomized, controlled trial, controlled trials, somatosensory, gerontology, geriatric, geriatrics, older adult, older adults, elder, elderly, older person, older people, ageing, aging, aged, game, games, gaming

## Abstract

**Background:**

Multidomain interventions have demonstrable benefits for promoting healthy aging, but self-empowerment strategies to sustain long-term gains remain elusive.

**Objective:**

This study evaluated the effects of digital somatosensory dance game participation on brain imagery changes as primary outcomes and other physical and mental health measures as secondary outcomes related to healthy aging.

**Methods:**

Between August 31, 2020, and June 27, 2021, this randomized controlled trial recruited 60 eligible participants older than 55 years with no recent engagement in digital dance games. A computer-generated randomization sequence was used to allocate participants 1:1, without stratification, to an intervention group (n=30) who underwent digital somatosensory dance game training or a control group (n=30). An anonymized code masked the intervention allocations from the investigators, and individuals who assigned the interventions were not involved in analyzing the study data. The intervention entailed two 30-minute dance game sessions per week for 6 months, and the control group received healthy aging education. Primary outcomes were brain imagery changes. All variables were measured at baseline and the 6-month follow-up, and intervention effects were estimated using *t* tests with intention-to-treat analyses.

**Results:**

Compared with the control group, intervention participants had significantly different brain imagery in the gray matter volume (GMV) of the left putamen (estimate 0.016, 95% CI 0.008 to 0.024; *P*<.001), GMV of the left pallidum (estimate 0.02, 95% CI 0.006 to 0.034; *P*=.004), and fractional amplitude of low frequency fluctuations of the left pallidum (estimate 0.262, 95% CI 0.084 to 0.439; *P*=.004). Additionally, the intervention group had different imagery in the cerebellum VI GMV (estimate 0.011, 95% CI 0.003 to 0.02; *P*=.01). The intervention group also had improved total Montreal Cognitive Assessment scores (estimate 1.2, 95% CI 0.27 to −2.13; *P*<.01), quality of life (estimate 7.08, 95% CI 2.35 to 11.82; *P*=.004), and time spent sitting on weekdays (estimate −1.96, 95% CI −3.33 to −0.60; *P*=.005). Furthermore, dance performance was significantly associated with cognitive performance (*P*=.003), health status (*P*=.14), resilience (*P*=.007), and demoralization (*P*<.001).

**Conclusions:**

Digital somatosensory dance game participation for 6 months was associated with brain imagery changes in multiple regions involving somatosensory, motor, visual, and attention functions, which were consistent with phenotypic improvements associated with healthy aging.

**Trial Registration:**

ClinicalTrials.gov NCT05411042; https://clinicaltrials.gov/study/NCT05411042

## Introduction

The process of aging manifests complex interplays between declining organ function, diminished physiological reserve, impaired functional performance, and the development of age-related health conditions. In 2015, the World Health Organization (WHO) published the “World Report on Aging and Health,” which proposed a function-centric, integrated approach to reforming the health and social care systems [1]; this paradigm shift is intended to enable health care systems to respond to the challenges of global population aging. To encourage the implementation of healthy aging principles internationally, the WHO launched its action plan “Decade of Healthy Aging” in 2020 [2]. The WHO defines “healthy aging” as “the process of developing and maintaining the functional abilities that enables wellbeing in older age” [2], which pursues healthy longevity by minimizing unhealthy years over the life course. A pragmatic, participatory, multidomain intervention for older community dwellers substantially improved their physical function, cognitive performance, nutritional status, and quality of life, even among people with multimorbidity [3,4]. With the success of identifying at-risk populations and validating the efficacy of intervention programs, limited research to date has affirmed consistent long-term gains in both physical and mental components [4]; however, the beneficial effects of interventions weaken as the frequency of supervised activities tapers off, possibly because of a lack of self-empowerment, especially during the COVID-19 pandemic [4].

“Gamification,” which incorporates pleasure and fun into health promotion activities, may contribute to sustaining beneficial intervention effects [5,6]. Digital somatosensory games are designed to engage participants in an interactive virtual environment that connects body movements with music and videos to achieve predefined goals [7]. Previous studies have reported that a gaming dance exergame is considered an enjoyable way for older adults to keep exercising. Playing digital somatosensory games not only includes physical activities but also involves sensory and cognitive coordination [8]. Hence, digital somatosensory games have been used in health care, rehabilitation, athletics, and other activities [9]. For example, Nintendo Wii Fit Plus games reportedly improve postural control and functional gait for frail older players [10], and nursing home residents who played digital somatosensory games were happier and more willing to exercise regularly [7]. Prior studies have also suggested that the use of digital games could promote structural and functional alterations in specific brain regions, such as the hippocampus, prefrontal cortex, and parietal lobe, leading to improvements in cognitive performance [11-13]. Additionally, other studies have shown that digital somatosensory games can substantially improve social, sensory, and emotional functions; physical activity levels; and disease prevention [8].

Theoretically, digital somatosensory dancing games represent suitable interventions to promote healthy aging in older adults because they integrate multitask activities that require extensive sensory coordination and are pleasurable [14]. However, no studies have evaluated the impact of digital somatosensory dance games with an explicit focus on brain signatures and healthy aging. Hence, this randomized controlled trial (RCT) aimed to study hypothetical benefits of digital somatosensory dance games on structural and functional brain imaging findings, cognitive performance, physical health, and mental health biomarkers.

## Methods

### Participants

**Figure 1:**
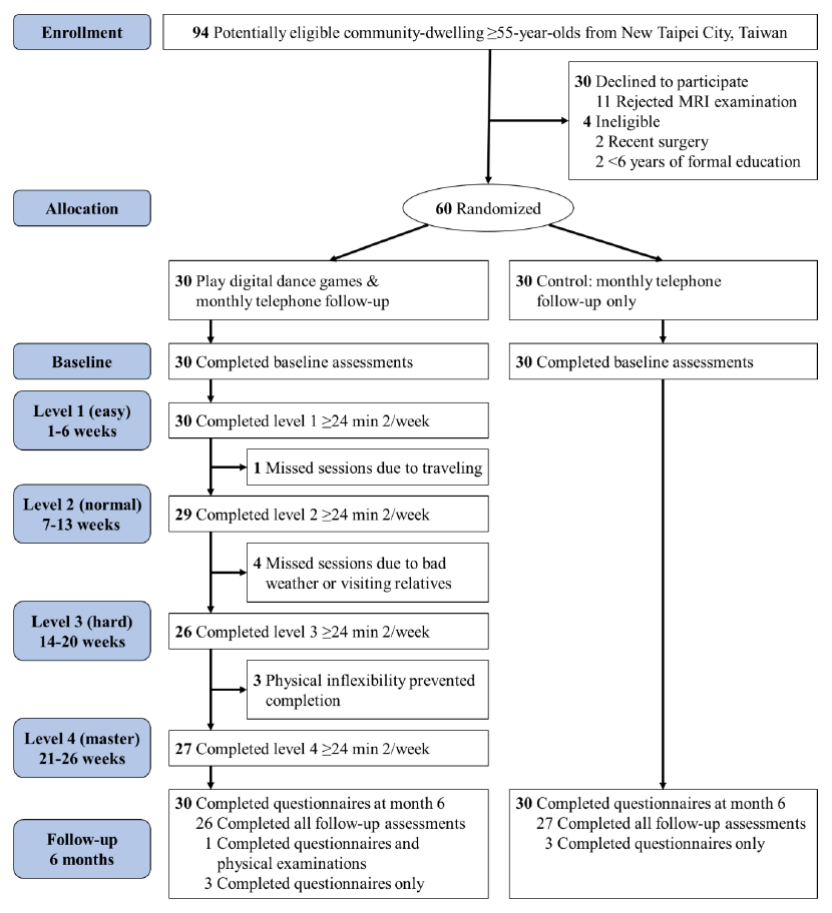
Study flowchart. MRI: magnetic resonance imaging.

This RCT enrolled community residents aged ≥55 years in Taipei, Taiwan, from August 31, 2020, to June 27, 2021, and followed them for 6 months; however, the trial was interrupted for approximately 3 months due to the COVID-19 pandemic (Figure 1). Participant inclusion criteria were as follows: (1) age ≥55 years, (2) ≥6 years of formal education, (3) spoke Mandarin and Taiwanese, (4) engagement in digital dance games <3 times per year during the past 3 years and not within the past 3 months, (5) ability to understand the research procedures and adhere to the prescribed study activities and assessments, and (6) provision of written informed consent. Participants meeting any of the following criteria were excluded from the study: (1) inability to communicate effectively with study staff, (2) undergoing active chemotherapy for malignancy, (3) life expectancy <12 months, and (4) presence of contraindications for magnetic resonance imaging (MRI) such as ferromagnetic foreign bodies or metal implants.

### Study Design and Participant Recruitment

This study was a single-blind, parallel-group RCT. Eligible participants were randomly allocated 1:1, without stratification, to either the intervention or control group according to a computer-generated random number sequence. Participants were not explicitly informed about their group assignment, and there was no contact between study investigators and participants during the study period. The intervention allocation was masked from investigators by anonymized coding, and the individuals who assigned interventions were not involved in assessing outcomes and analyzing data. The investigators also avoided any potential interactions between participants in either group. The participants were recruited from the neighborhood community, and flyers were posted in neighborhood health centers and local district service offices. The content of the flyer included the research purpose, health examination results, and compensation information.

### Staff Training

All researchers completed education workshops before the study began using a written instruction manual to ensure standard implementation of study interventions.

### Ethical Considerations

#### Human Subject Ethics Review and Approvals

This study was approved by the institutional review board of National Yang Ming Chiao Tung University (YM108123F).

#### Informed Consent

Informed consent was obtained, and the right of participants to withdraw from the program was explained.

#### Privacy and Confidentiality

The electronic data for analysis were deidentified, and the questionnaires with the participants’ signatures were stored in a locked place to safeguard participant information.

#### Compensation Details

The participants received breakfast following their morning fasting blood work and a gift card worth NT$100 (US $3.30) as compensation during both the baseline and posttest phases.

### Procedures

#### Baseline Measurements

Before the first dance game session, participants in both study arms underwent 3-T brain MRI scans and completed questionnaires on physical activity, resilience, demoralization, and quality of life; baseline measurements included physical performance, body composition, and blood biochemistry biomarkers.

#### Digital Somatosensory Dance Game Intervention

The intervention group played a digital somatosensory dance game (DANZ BASE; International Games System Company) at a community health center; these sessions, each lasting ≥30 minutes (including a 5-7–minute warm-up and cooldown), took place twice weekly for 6 months.

In each digital somatosensory dance game session, participants danced to 3 songs categorized as 1, 2, or 3 stars according to the increasing complexity of the dance movements. Each of these songs also had 4 levels of difficulty (1: easy; 2: ordinary; 3: hard; 4: master), which were used in successive intervention periods. DANZ-BASE has standard levels of interactive dance movements for each song, and after completing each song, participants are scored based on the accuracy with which their dance movements followed those of the on-screen virtual performers. An integrated digital camera evaluates the dance movements of performers at 4 predefined levels (miss, nice, great, and perfect) based on the timing and positioning of participants compared with the images on the screen (Tables S1-S3 and Figure S1 in Multimedia Appendix 1).

The scheduled 6-month program was modified to mitigate withdrawals from the study, especially during the COVID-19 pandemic, which could have potentially lessened observable intervention effects. Notably, the intervention had to be interrupted at difficulty level 4 for 1 month due to the pandemic, and the remaining sessions were recommenced at level 3 or level 2.

Control group participants underwent the same assessments and examinations at baseline and at the end of the study as the intervention group, including the questionnaire, physical examination, laboratory tests, and MRI (Table S1 in Multimedia Appendix 1); however, during the 6-month intervention period, they only received healthy aging education fact sheets and monthly telephone calls to monitor their daily activities.

We tried to enhance adherence in several ways, including assigning participants a dedicated research assistant who contacted them monthly by telephone to determine if they had any difficulties with adhering to the program and to help resolve any issues as needed.

### Outcomes and Measurements

#### Primary Outcome: Neuroimaging

The primary outcome was brain imagery changes as shown on neuroimaging.

All participants underwent 2 MRI brain scans. The first scan used a Siemens 3T MAGNETOM Trio scanner; the second MRI scan was done with an upgraded Siemens MAGNETOM Prisma^fit^ system, which includes enhanced gradient performance and upgraded software. Both Siemens scanner systems are widely used in neuroimaging studies. Although MRI scanner upgrades may introduce system effects in acquired images, we did not change the main static MRI magnet and sought to minimize image acquisition effects. Whole-brain T1-weighted anatomical data from both MRI scan sessions were acquired using a 3-dimensional magnetization-prepared rapid-acquisition gradient-echo (3D-MPRAGE) sequence without in-plane interpolation and interslice gap with the following parameters: TR (repetition time)/TE (echo time)/TI (inversion time)=3500/3.5/1100 ms; flip angle=7°; field of view=256 × 256 mm^2^; matrix size=256 × 256; voxel size=1.0 × 1.0 × 1.0 mm^3^; number of excitations=1; number of sagittal slices=192. Whole-brain resting-state functional MRIs were acquired using an axial T2*-weighted gradient-echo echo planar imaging sequence with the following imaging parameters: TR/TE=2500/27 ms; flip angle=77°, field of view=220 × 220 mm^2^; image matrix=64 × 64; slice thickness=3.4 mm; 200 continuous image volumes; 43 interleaved partitions without an interpartition gap. An experienced researcher visually assessed all anatomical scans for image quality to exclude images with brain trauma, tumors, hemorrhagic or infarct lesions, or severe motion artifacts.

To investigate potential brain signature alterations associated with digital dance game training, we estimated the individual voxel-wise brain maps, including gray matter volume (GMV), regional homogeneity (ReHo), fractional amplitude of low frequency fluctuations (fALFF), and global brain connectivity (GBC). During image preprocessing of structural data, we used the enhanced Diffeomorphic Anatomical Registration Through Exponentiated Lie Algebra voxel-based morphometry (DARTEL-VBM) procedure to generate individual GMV maps. Image processing was done using Statistical Parametric Mapping (SPM12, version 7487; Wellcome Institute of Neurology) software using MATLAB (R2020a, Mathworks). Details of the enhanced DARTEL-VBM framework are described in our previous studies [15-17]. Briefly, image processing of T1-weighted images entails (1) bias-field correction, (2) 3 tissue-type segmentations (gray matter, white matter, and cerebrospinal fluid), (3) spatial normalization to a study template in the Montreal Neurological Institute (MNI) space, (4) tissue modulation for the GMV map, and (5) spatial smoothing with a 6-mm Gaussian kernel. Finally, we estimated the smoothed GMV voxel-wise map, which represents the brain structure.

During image preprocessing of resting-state functional MRI (rsfMRI) data, all rsfMRI image processing was performed using a combination of the Analysis of Functional Neuroimaging (AFNI, version 20.3.03) software package, the Functional Magnetic Resonance Imaging of the Brain Software Library (FSL, version 5.0.10), and SPM12. The preprocessing of the individual rsfMRI data sets was carried out according to the methods outlined in our previous study [18], and the pipeline included the following steps: (1) removal of the first 10 volumes for steady‐state magnetization; (2) motion correction for functional volume alignment; (3) slice timing correction to correct the temporal misalignment during image acquisition across slices; (4) brain extraction to remove nonbrain tissue; (5) despiking and detrending to remove the spike artifact and polynomial trend; (6) linear regression to remove nuisance signals including Friston 24-motion parameters, white matter, and cerebrospinal fluid signals; (7) spatial normalization and resampling to the standard MNI space with a 2-mm isotropic voxel size. Subsequently, we calculated 3 functional measurements through appropriate analyses, including ReHo, fALFF, and GBC. These measurements represent aspects of spontaneous brain activity and brain architecture.

The calculation of ReHo involved assessing regional synchronization using the Kendall coefficient of concordance (KCC) for a voxel's time series in conjunction with its nearest 26 neighboring voxels [19]. Prior to ReHo computation, bandpass temporal filtering (0.01-0.08 Hz) was applied to mitigate low-frequency drift, high-frequency noise, and nonneural signals. Individual ReHo maps were generated by computing the KCC for each voxel and subsequently normalizing them by subtracting the mean value of the entire brain and dividing by the standard deviation, thereby producing standardized ReHo maps. Finally, these resultant maps were subjected to spatial smoothing using a 6-mm full-width at half-maximum (FWHM) Gaussian kernel, resulting in individual, smoothed ReHo voxel-wise maps.

In the process of measuring fALFF, the preprocessed functional data were smoothed with a 6‐mm FWHM Gaussian kernel [20]. Subsequently, the time series of each voxel was converted to the frequency domain, and the power spectrum was computed using fast Fourier transform. Voxel-wise fALFF maps were then generated by determining the fraction of power within the specific low-frequency band (0.01-0.08 Hz) relative to the power spectrum amplitudes across the entire frequency range (0-0.2 Hz). Following this calculation, the fALFF maps were normalized by subtracting the average value and dividing by the standard deviation, resulting in individualized, normalized fALFF voxel-wise maps.

Before estimating the GBC measurements, the preprocessed images underwent a bandpass temporal filter and spatial smoothing using a 6-mm FWHM Gaussian kernel [21]. The images were then resampled to 3-mm voxel size, and a gray matter mask was applied to reduce computational load. Subsequently, the individual GBC voxel-wise maps were generated by computing the correlation between each gray matter voxel and all other gray matter voxels. These correlations were then transformed into Fisher *z* scores and averaged.

Furthermore, the whole brain GMV, ReHo, fALFF, and GBC voxel-wise maps were derived by averaging values across various brain regions defined in a composite brain atlas. This atlas comprised 110 cortical regions sourced from the Harvard-Oxford template [22] and 27 cerebellar regions from a spatially unbiased infratentorial template [17]. Subsequently, these averaged brain signatures for each participant were used in subsequent group-level statistical analyses.

#### Secondary Outcomes

Secondary outcomes included cognitive performance, physical health (physical activity, body composition, and physical performance), mental health (resilience, demoralization, and quality of life), and related biomarkers (hematology and urinalysis).

Cognitive performance was evaluated using the Montreal Cognitive Assessment (MoCA) [23]; the MoCA contains 8 subdomains: (1) orientation, (2) short-term memory and delayed recall, (3) executive function and visuospatial ability, (4) language skills, (5) abstract thinking, (6) verbal fluency, (7) attention, and (8) clock drawing test. The MoCA score ranges from 0 to 30, with ≥26 considered normal [24].

The International Physical Activity Questionnaire (IPAQ) [25] was used to assess physical activity, which includes working, household chores, commuting, and exercise. Greater scores indicate greater physical activity. IPAQ has been used in many countries with a content validity index of 0.994 and a test-retest reliability of 0.67 for the short-form version [25,26].

Resilience was measured using the Brief Resilience Scale (BRS), which is a short, self-administered questionnaire that assesses respondents’ abilities to recover from stress and adversity [27]. The BRS has 6 questions to elicit responses on a 5-point Likert-type scale, ranging from 1 (strongly disagree) to 5 (strongly agree). Responses to negatively phrased questions are reverse scored, and the mean score of all 6 items constitutes the overall resilience status, with higher scores indicating better resilience [27].

The Demoralization Scale-Mandarin Version that was used to measure demoralization comprises 24 items, each rated on a 5-point Likert scale, ranging from 0 (never) to 4 (all the time) [28,29]; a cut-off score of ≥30 is considered to affirm demoralization [28]. The German version sets cut-offs of 30 to indicate moderate demoralization and 36 to signify a high level of demoralization in patients with advanced cancer [30].

Quality of life was evaluated using the EQ-5D questionnaire, which assesses 5 domains: mobility, self-care, pain, usual activities, and psychological status [31]. Responses are categorized as grade 1 (no problem), 2 (moderate problem), or 3 (severe problem) [31]. The EQ-5D also includes a visual analogue scale from 0 to 100, with a higher score indicating better general health status [32].

#### Other Variables

Other study data included demographic characteristics, health behaviors (including tobacco or alcohol use), physical performance, and self-reported physician-diagnosed medical conditions.

### Statistical Analysis

Based on a previous study of somatosensory games with older adults [33], we used a functional connectivity gaming comparison before and after data collection. By inputting the samples of the 2 groups (45 and 42) and the *t* value (–4.00) into a Cohen *d* calculation, the resulting Cohen *d* was –0.8582. This value was entered into G-power with a *t* test. The difference between the 2 independent means (2 groups), with a 2-tailed effect size of –0.8582 and power of 0.9, resulted in a total sample size of 60 (30 in each group).

Numerical variables are presented as the mean (SD). Discrete variables are presented as the number (percentage). Student *t* tests were used to compare the changes between pre- and postintervention, demonstrating effects of the intervention on the primary and secondary outcomes. Intention-to-treat analysis was used with the Student *t* test and mean values to manage missing data under the original RCT design. In the intervention group, associations between the study intervention and secondary outcomes were estimated using a stepwise model in regression analysis. Dance intervention performance was measured using the completion and correction rates. In the primary outcomes, we first conducted statistical analysis on the averaged GMV, then explored the statistical potential changes of functional signatures (fALFF, ReHo, and GBC) in significant structural brain regions.

A 2-sided, uncorrected *P* value <.05 and 95% CIs not spanning the null hypothesis value were considered statistically significant. All analyses were performed using SPSS Windows, version 25.0 (IBM Corp). In addition, the threshold of significance was set at *P*<.05 corrected by the false discovery rate (FDR) for multiple comparisons. Finally, to further investigate the relationship between regional GMV alterations and cognitive performance in the intervention group, exploratory partial Pearson correlation analyses were then used to examine the associations between regional GMV differences and cognitive performance difference (total MoCA score and the 8 subdomain scores), with age, sex, and total intracranial volume serving as nuisance variables. The significance threshold for the partial Pearson correlation analyses was set at an uncorrected *P*<.05.

## Results

### Participants

From August 31, 2020, to June 27, 2021, 94 potential participants were screened for eligibility, 60 of whom met the inclusion criteria and were enrolled and randomized (Figure 1). Table 1 shows the baseline characteristics of each study arm. Both groups had a mean age of approximately 68 years (intervention: 68.3, SD 5.1 years; control: 68.2, SD 5.5 years), and women represented 97% (29/30) of the intervention group and 60% (18/30) of the control group.

**Table 1:** Baseline participant characteristics.

Characteristics		Intervention group (n=30)	Control group (n=30)
Age (years), mean (SD)		68.3 (5.1)	68.2 (5.5)
**Sex, n (%)**			
	Female	29 (97)	18 (60)
	Male	1 (3)	12 (40)
Education (years), mean (SD)		12.2 (3.0)	14.1 (3.6)
**Employment status, n (%)**			
	Full-time	2 (7)	4 (13)
	Part-time	6 (20)	1 (3)
	Homemaker	2 (7)	2 (7)
	Retired	20 (66)	23 (77)
**Alcohol consumption, n (%)**			
	Lifelong abstainer	9 (30)	6 (20)
	Former drinker	18 (60)	10 (33)
	Current drinker	3 (10)	14 (47)
**Tobacco smoking, n (%)**			
	Never	29 (97)	23 (77)
	Former smoker	1 (3)	7 (23)
	Current smoker	0	0
**Immunization status, n (%)**			
	Influenza	10 (33)	17 (57)
	Pneumococcus	10 (33)	14 (47)
	Varicella zoster	2 (7)	3 (10)
	COVID-19	26 (87)	27 (90)
**Medication use, n (%)**			
	Pharmaceutical agents	16 (53)	21 (70)
	Health supplements	22 (73)	19 (63)

The completion rate in the intervention group was influenced by the difficulty level of dance movements and the participation rate. The easiest level (level 1) was used for the first 6 weeks, with a completion rate of 100%; however, due to the COVID-19 pandemic, participation and completion rates later fell below 100%. Table S1 in Multimedia Appendix 1 presents the details and the rationale for the 4 participants who failed to complete the scheduled intervention.

### Primary Outcome

**Figure 2:**
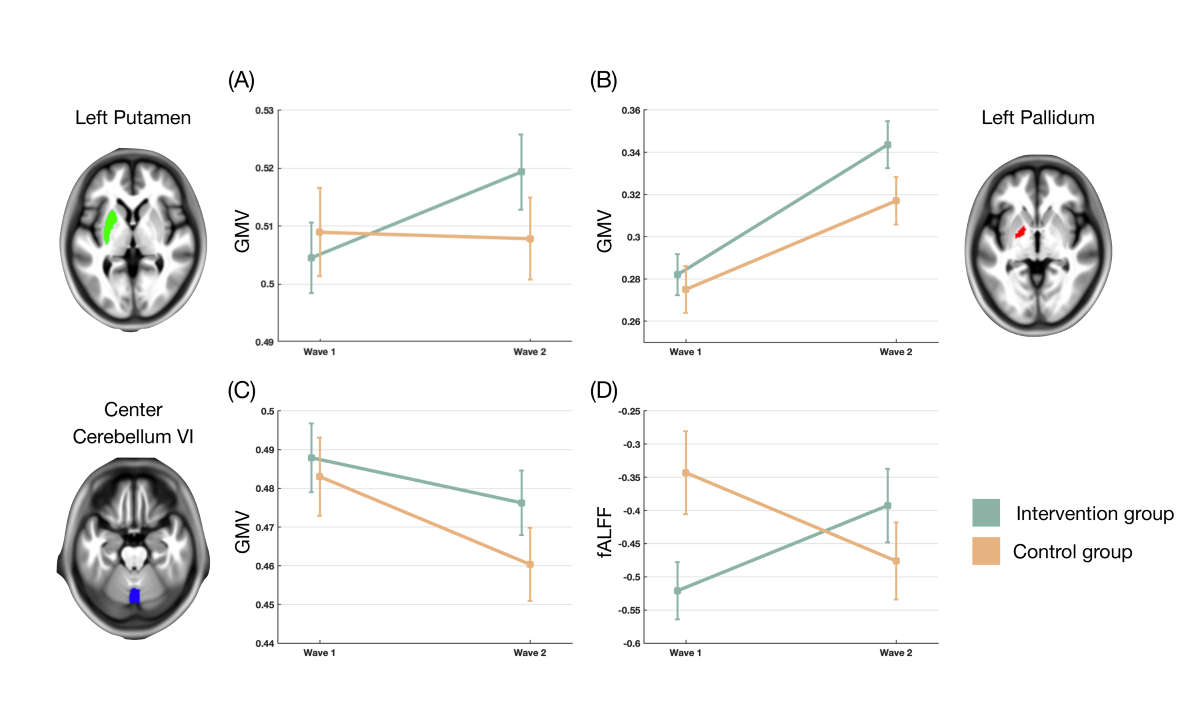
Comparison between the intervention and control groups of the slopes of the (A) left putamen gray matter volume (GMV), (B) left pallidum GMV, (C) center cerebellum VI GMV, and (D) left pallidum fractional amplitude of low frequency fluctuations (fALFF).

During this study, intervention participants had significantly different brain imagery of the left putamen GMV (estimate 0.016, 95% CI 0.008 to 0.024; *P*<.001, FDR-corrected *P*=.14), left pallidum GMV (estimate 0.02, 95% CI 0.006 to 0.034; *P*=.004, FDR-corrected *P*=.27), left pallidum fALFF (estimate 0.262, 95% CI 0.084 to 0.439; *P*=.004, FDR-corrected *P*=.14), and cerebellum VI GMV (estimate 0.011, 95% CI 0.003 to 0.02; *P*=.01, FDR-corrected *P*=.38; Table 2). Additionally, the intervention participants had a significant change in the GMV and GBC of the left putamen, GMV and fALFF of the left pallidus, and GMV and ReHo of the center cerebellum VI (Table 2). Figure 2 shows that, compared with the control group, the intervention group exhibited a significant increase in the slope of the left putamen GMV and left pallidus GMV and a notable decrease in the downward slope of the center cerebellum VI GMV. Furthermore, there was a significant difference in the diagonal trajectory of the left pallidus fALFF between the intervention and control groups. Figure S2 exhibits the significant relationships between the change in the left pallidum GMV and total MoCA score (*r*=–0.393, *P*=.04) and abstract thinking subdomain score of the MoCA (*r*=–0.531, *P*=.004).

**Table 2:** Changes in brain regions of interest between the intervention and control groups.

Variables			Intervention (n=30)									Control (n=30)									Intervention effect^a^					FDR^b^-corrected *P* value
			Baseline (T0), mean (SD)		6 months (T1), mean (SD)		T1-T0		*P* value		Baseline (T0), mean (SD)			6 months (T1), mean (SD)		T1-T0		*P* value		Estimate (95% CI)			*P* value			
**Left putamen**																										
	GMV^c^	0.50 (0.03)		0.52 (0.04)		0.01 (0.01)		<.001		0.51 (0.04)			0.51 (0.04)		0.00 (0.02)		.74		0.016 (0.008 to 0.024)			<.001		.14		
	ReHo^d^	–0.24 (0.19)		–0.26 (0.25)		–0.02 (0.20)		.56		–0.12 (0.28)			–0.22 (0.24)		–0.10 (0.23)		.02		0.079 (–0.028 to 0.185)			.15		.67		
	fALFF^e^	–0.01 (0.26)		0.04 (0.36)		0.05 (0.29)		.39		0.17 (0.39)			0.12 (0.35)		–0.05 (0.33)		.39		0.100 (–0.056 to 0.255)			.21		.65		
	GBC^f^	0.31 (0.02)		0.32 (0.02)		0.01 (0.02)		.03		0.32 (0.03)			0.32 (0.02)		0.00 (0.02)		.81		0.006 (–0.003 to 0.016)			.18		.59		
**Left pallidum**																										
	GMV	0.28 (0.05)		0.34 (0.06)		0.06 (0.03)		<.001		0.28 (0.06)			0.32 (0.06)		0.04 (0.03)		<.001		0.020 (0.006 to 0.034)			.004		.27		
	ReHo	–0.59 (0.20)		–0.58 (0.24)		0.00 (0.21)		.96		–0.48 (0.24)			–0.57 (0.23)		–0.09 (0.25)		.06		0.092 (–0.023 to 0.208)			.12		.67		
	fALFF	–0.52 (0.23)		–0.39 (0.30)		0.13 (0.30)		.03		–0.34 (0.34)			–0.48 (0.32)		–0.13 (0.41)		.08		0.262 (0.084 to 0.439)			.004		.14		
	GBC	0.16 (0.01)		0.16 (0.01)		0 (0.01)		.23		0.15 (0.01)			0.15 (0.01)		0.00 (0.01)		.74		0.002 (–0.003 to 0.007)			.44		.73		
**Center cerebellum VI**																										
	GMV	0.49 (0.05)		0.48 (0.05)		–0.01 (0.01)		<.001		0.48 (0.06)			0.46 (0.05)		–0.02 (0.02)		<.001		0.011 (0.003 to 0.02)			.01		.38		
	ReHo	–0.31 (0.25)		–0.20 (0.26)		0.10 (0.19)		.005		–0.11 (0.28)			0.11 (0.33)		0.21 (0.29)		<.001		–0.110 (–0.232 to 0.012)			.08		.56		
	fALFF	–0.32 (0.42)		–0.26 (0.31)		0.06 (0.35)		.37		–0.20 (0.32)			–0.11 (0.34)		0.09 (0.43)		.24		–0.035 (–0.229 to 0.160)			.73		.91		
	GBC	0.37 (0.01)		0.38 (0.01)		0.01 (0.02)		.14		0.38 (0.02)			0.38 (0.03)		0.01 (0.03)		.08		–0.003 (–0.014 to 0.008)			.54		.76		

^a^Intervention effect computed as the between-group difference in mean change from baseline to month 6 (change in intervention group minus change in control group).

^b^FDR: false discovery rate.

^c^GMV: gray matter volume.

^d^ReHo: regional homogeneity.

^e^fALFF: fractional amplitude of low-frequency fluctuations.

^f^GBC: global brain connection.

### Secondary Outcomes

Table 3 also shows the changes in cognitive performance, physical activity, resilience, demoralization, and quality of life, together with estimates of the intervention effects. There were significant postintervention between-group differences in total MoCA (estimate 1.2, 95% CI 0.27 to −2.13; *P*<.01, FDR-corrected *P*=.08), time spent sitting on weekdays (estimate −1.96, 95% CI −3.33 to −0.60; *P*=.005, FDR-corrected *P*=.053), and quality of life (estimate 7.08, 95% CI 2.35 to 11.82; *P*=.04, FDR-corrected *P*=.053). The MoCA abstract thinking subdomain score significantly improved from baseline to 6 months in the intervention group but not in the control group; the difference between the groups was not statistically different. For body composition and physical performance, body weight decreased in the intervention group. In addition, body muscle mass, muscle mass of both legs, skeletal muscle mass, and calories expended per day decreased in both groups. For biomarkers, mean corpuscular volume (estimate −1.20, 95% CI −2.21 to −0.20; *P*=.02, FDR-corrected *P*=.11) and thyroid-stimulating hormone (estimate −0.69, 95% CI −1.11 to −0.28; *P*=.002, FDR-corrected *P*=.053) were different between the groups.

**Table 3:** Comparisons of changes in secondary outcomes between the intervention and control groups.

Outcomes		Intervention group (n=30)			Control group (n=30)				Intervention effect^a^			Corrected *P* value^b^
		Baseline, mean (SD)	6 months, mean (SD)	*P* value	Baseline, mean (SD)	6 months, mean (SD)	*P* value	Estimate (95% CI)		*P* value		
**Cognitive performance (MoCA^c^)**												
	Total score	26.03 (2.66)	27.42 (2.68)	.001	26.63 (2.22)	26.81 (2.69)	.50	1.20 (0.27 to 2.13)		.01	.08	
	Verbal fluency (naming)	2.65 (0.58)	2.80 (0.53)	.06	2.96 (0.18)	3.00 (0)	.27	0.12 (–0.06 to 0.29)		.18	.65	
	Abstract thinking	1.42 (0.54)	1.73 (0.50)	.003	1.63 (0.53)	1.85 (0.34)	.005	0.09 (–0.15 to 0.32)		.47	.79	
**Physical activity (IPAQ^d^)**												
	Time spent sitting on weekdays (hours)	2.92 (2.25)	1.37 (2.18)	.002	2.44 (3.17)	2.85 (3.18)	.44	–1.96 (–3.33 to –0.60)		.005	.053	
	MET^e^ (kcal per week)	2419.26 (1763.94)	2393.41 (1368.57)	.90	1814.36 (1006.39)	1885.38 (1326.06)	.68	–96.87 (–620.58 to 426.84)		.71	.88	
	5 times sit to stand test (second)	7.03 (1.71)	7.14 (1.53)	.68	8.91 (2.69)	9.65 (2.68)	.18	–0.63 (–1.84 to 0.59)		.31	.70	
Resilience (BRS^f^ score)		4.05 (0.45)	4.16 (0.51)	.09	3.80 (0.63)	3.89 (0.51)	.36	0.03 (–0.20 to 0.26)		.80	.89	
Demoralization (total DS-MV^g^ score)		16.65 (10.05)	17.57 (10.51)	.50	21.74 (12.23)	21.55 (9.99)	.89	1.11 (–2.63 to 4.85)		.56	.79	
**Quality of life (EQ-5D)**												
	Visual analog score	76.73 (9.82)	81.50 (10.09)	.02	78.06 (8.56)	75.74 (8.80)	.11	7.08 (2.35 to 11.82)		.004	.053	
**Body composition and physical performance**												
	Body weight (kg)	58.72 (7.84)	57.76 (7.71)	.007	63.73 (8.46)	63.06 (8.79)	.09	–0.30 (–1.31 to 0.72)		.56	.79	
	Body mass index (kg/m^2^)	24.35 (3.35)	23.97 (3.29)	.005	24.09 (2.57)	23.92 (2.66)	.23	–0.20 (–0.57 to 0.17)		.29	.70	
	Body fat mass (kg)	19.28 (5.24)	18.87 (5.15)	.23	18.07 (5.32)	17.98 (5.09)	.85	–0.32 (–1.49 to 0.86)		.59	.79	
	Body fat (%)	32.40 (5.62)	32.20 (5.40)	.58	28.29 (6.88)	28.38 (6.21)	.87	–0.30 (–1.68 to 1.07)		.66	.85	
	Body muscle mass (kg)	21.22 (2.75)	20.93 (2.61)	.004	25.28 (4.86)	24.62 (4.25)	.12	0.38 (–0.47 to 1.23)		.38	.79	
	Right leg muscle mass (kg)	5.88 (0.83)	5.76 (0.83)	.008	7.13 (1.35)	6.90 (1.34)	<.001	0.05 (–0.09 to 0.19)		.50	.79	
	Left leg muscle mass (kg)	5.83 (0.78)	5.72 (0.78)	.005	7.06 (1.27)	6.87 (1.27)	<.001	0.09 (–0.02 to 0.19)		.12	.49	
	Handgrip strength (kg)	25.54 (4.77)	25.38 (4.81)	.64	28.11 (6.37)	29.09 (7.19)	.13	–1.14 (–2.57 to 0.29)		.12	.49	
	Knee height (cm)	45.75 (2.14)	45.50 (2.22)	.19	48.89 (2.10)	47.98 (1.97)	<.001	0.66 (0.14 to 1.19)		.01	.08	
	Corrected vision of right eye	0.56 (0.21)	0.59 (0.22)	.26	0.66 (0.26)	0.73 (0.24)	.03	–0.04 (–0.11 to 0.03)		.29	.70	
	Skeletal muscle index (kg/m2)	6.39 (0.76)	6.31 (0.78)	.02	7.00 (1.13)	6.90 (1.11)	.008	0.01 (–0.08 to 0.10)		.78	.89	
	Phase angle (rad)	4.96 (0.42)	4.86 (0.47)	.04	5.11 (0.45)	4.97 (0.43)	.01	0.05 (–0.09 to 0.19)		.50	.79	
	Bone mass index (gm/cm2)	2.33 (0.25)	2.28 (0.23)	<.001	2.65 (0.42)	3.30 (3.22)	.30	0.01 (–0.08 to 0.10)		.78	.89	
	Body moisture content (kg)	28.95 (3.37)	28.54 (3.19)	.001	33.53 (5.42)	33.11 (5.23)	.03	0.42 (–12.58 to 13.41)		.95	.98	
	Burn calories per day (kcal)	1221.88 (98.53)	1210.19 (93.60)	.002	1356.04 (158.98)	1343.93 (153.20)	.04	0.01 (–0.42 to 0.46)		.95	.98	
	Right foot resistance (Ohm)	201.05 (26.71)	206.99 (29.32)	.04	187.60 (33.30)	197.30 (33.18)	<.001	–3.75 (–10.55 to 3.04)		.27	.70	
	Left foot resistance (Ohm)	204.70 (25.89)	210.85 (29.56)	.02	189.50 (30.43)	198.17 (32.23)	<.001	–2.52 (–8.85 to 3.81)		.43	.79	
**Hematology and urinalysis**												
	Mean corpuscular volume (fL)	94.10 (3.64)	93.20 (3.64)	.005	93.63 (6.23)	93.93 (6.36)	.47	–1.20 (–2.21 to –0.20)		.02	.11	
	Alanine aminotransferase (U/L)	30.50 (27.65)	26.31 (26.24)	.004	26.89 (17.48)	22.70 (8.11)	.09	–0.01 (–5.51 to 5.50)		≥.99	≥.99	
	Homocysteine (umol/L)	15.18 (3.23)	12.52 (2.67)	<.001	16.65 (7.41)	14.66 (4.77)	.02	–0.68 (–2.70 to 1.34)		.50	.79	
	Albumin to creatinine ratio	10.77 (10.32)	12.15 (17.60)	.62	24.66 (84.97)	28.95 (107.48)	.31	–2.90 (–12.93 to 7.11)		.56	.79	
	High-density lipoprotein (mg/dL)	60.80 (10.61)	56.50 (9.40)	.003	59.44 (11.06)	57.40 (10.81)	.15	–2.27 (–6.08 to 1.54)		.24	.70	
	Thyroid-stimulating hormone (uIU/mL)	2.42 (1.28)	2.05 (1.08)	.004	1.87 (1.15)	2.19 (1.25)	.07	–0.69 (–1.11 to -0.28)		.002	.053	

^a^Intervention effect computed as the between-group difference in mean change from baseline to month 6 (change in intervention group minus change in control group).

^b^Corrected for the false discovery rate.

^c^MoCA: Montreal Cognitive Assessment.

^d^IPAQ, International Physical Activity Questionnaire.

^e^MET: metabolic equivalent.

^f^BRS: Brief Resilience Scale.

^g^DS-MV: Demoralization Scale Mandarin Version.

　　Hierarchical regression analyses (Table 4) showed that dancing performance (completion rate of the intervention for >24 minutes per session) was independently associated with cognitive performance (β=0.647, *P*=.21) and self-rated health status on the IPAQ (β=1.159, *P*=.008). Moreover, the correct rate for the assigned dance at a difficulty level of 3 was related with resilience (β=–5.629, *P*=.007) and demoralization (β=−0.251, *P*<.001).

**Table 4:** Associations between dance intervention performance and secondary outcomes (n=30).

Outcomes		Unstandardized B (SE)	Standardized β	*t* test (*df*)	*P* value
**Increased cognitive function (MoCA^a^)**					
	Completion rate of interventions for >24 minutes per session	0.647 (0.264)	0.394	2.453 (4)	.02
	Correct rate for assigned dance with level 2 difficulty	0.937 (0.356)	0.420	2.633 (4)	.01
	Correct rate for assigned dance with level 3 difficulty	0.955 (0.295)	0.417	3.237 (4)	.003
**Increased self-rated daily health status (EQ-VAS^b^)**					
	Completion rate of interventions for >24 minutes per session	–1.159 (0.399)	–0.455	–2.905 (4)	.008
	Correct rate for assigned dance with level 3 difficulty	–1.177 (0.446)	–0.331	–2.638 (4)	.01
**Affecting increased resilience (BRS^c^)**					
	Correct rate for assigned dance with level 3 difficulty	–5.629 (1.903)	–0.415	–2.958 (4)	.007
**Affecting decreased demoralization level (DS-MV^d^)**					
	Correct rate for assigned dance with level 1 difficulty	–0.382 (0.124)	–0.622	–3.081 (4)	.005
	Correct rate for assigned dance with level 2 difficulty	–0.370 (0.105)	–0.628	–3.529 (4)	.002
	Correct rate for assigned dance with level 3 difficulty	–0.351 (0.087)	–0.579	–4.030 (4)	<.001

^a^MoCA: Montreal Cognitive Assessment.

^b^VAS: visual analogue scale.

^c^BRS: Brief Resilience Scale.

^d^DS-MV: Demoralization Scale Mandarin Version.

## Discussion

### Principal Findings

　　To the best of our knowledge, this is the first RCT to substantiate beneficial effects of playing digital somatosensory dance games on measures of healthy aging, especially concerning neuroimaging findings. The results support the hypothesis that participating in digital somatosensory dance games not only results in favorable changes in brain structures and function in the putamen, pallidum, and cerebellum but also promotes cognitive performance, daily physical activity, healthier body composition, and quality of life.

The unique strength of this study was the robust primary outcome of brain signatures, which have not been previously reported. The clinical benefits of exergames or somatosensory games in previous studies were shown among small samples in case-control designs with relatively short intervention periods, questionnaires and interview-based assessments for outcomes, and neither biomarker measurements nor brain signatures. Our study showed evident clinical benefits of digital somatosensory dance game participation on cognitive performance, and favorable brain signature changes strongly support the biological effects of the intervention, despite the intervention activities being interrupted by the COVID-19 pandemic.

### Comparison With Prior Work

　　Digital somatosensory dance game training entails visual observation and physical practice to acquire motor and coordination skills; this involves the integration of multiple cognitive domains, including somatosensory, motor, visual, attention, executive function, and reward processing. Previous studies have shown that these cognitive functions involve multiple brain regions, including the medial prefrontal, cingulate, premotor, parietal, and occipitotemporal cortices [34]. Our results show that digital somatosensory dance game training significantly increases structural and functional characteristics in the left putamen GMV, left pallidum GMV, center cerebellum VI GMV, and left pallidum fALFF. Pertinently, these brain regions are linked to higher-order cognitive functions, such as somatosensory, attention, episodic memory, and motor imagery [35].

　　Most video games include rewarding effects to increase the motivation of players to continue to participate. Such rewards may substantially alter the reward system, either functionally or structurally, in brain regions that include the striatum, pallidum, and cingulate cortex [36]. Consistent with reported reward-related effects, we also observed GMV increases in the putamen and pallidum among intervention participants; notably, we detected significant changes in the cerebellum VI, which controls motor functions and is also involved in visual working memory, verbal fluency, creativity, and cognition-related activities [37,38]. Manifest brain structure changes not only support phenotypic improvements in physical and cognitive functions but are also relevant to higher-order cognitive function. Our previous research showed that the cerebellum-limbic neurocircuit plays significant roles in the development of physiocognitive decline syndrome, which is a key phenotype associated with unhealthy aging [7]. The results of this RCT and another recent study [39] support considering the cerebellum-limbic connection as a biosignature of healthy aging. Besides benefiting physical activities and physical performance, playing the digital somatosensory dance game for 6 months significantly preserved brain regions implicated in reward processing.

　　Although digital dance games were originally developed for youth entertainment, they can be adapted to improve the physical and mental health of older adults [5]. The favorable effects of participating in digital somatosensory dance games support expanding their applications to promoting healthy aging rather than merely entertaining youngsters. People playing such games must perform complex and rhythmic dance moves that entail extensive coordination of visual and auditory functions and active body movements. Adcock et al [5] evaluated an exergame that incorporated tai chi and dance exercises, showing positive associations between physical activities and functioning in a small sample. Others have demonstrated the feasibility of unsupervised sensor-based exercise (step mat training and Microsoft Kinect) at home for older adults, which significantly reduced the likelihood of falls and improved cognitive function [40]. Previous research has shown that playing somatosensory games may contribute to improving cognitive function; however, which domains of cognitive function are affected has not been firmly established [6,7].

In the aging process, domain-specific declines in cognitive function suggest different trajectories of aging, with verbal, language, and executive function domains being both the earliest to decline over time and potentially reversible to promote healthy aging [41]. Improvements in physical and cognitive performance and in brain structural images associated with participating in digital somatosensory dance games are consistent with the aforementioned studies. Importantly, there was a statistically significant relationship between the rate of completing somatosensory dance game levels and improved cognitive function.

Although resilience and demoralization were not significantly improved after the intervention, dance performance was associated with resilience and demoralization. Notably, psychosocial health is an important aspect of healthy aging, and previous studies have identified demoralization and resilience as factors that are associated with good quality of life [42,43]. A lack of a statistically significant intervention effect on resilience and demoralization may be explained by ceiling effects; participants had a good quality of life, good resilience, and low demoralization levels. Nevertheless, the pleasure derived from playing the digital somatosensory dance game is important for both mental well-being and for adherence to and participation in health promotion activities. Furthermore, game-playing also provides alternatives to augment conventional multidomain intervention programs that increase the diversity of activities and ensure their effectiveness. Motor-cognitive dual-task training has been shown to improve the physical and cognitive performance of people with various neurodegenerative disorders [44,45], and digital somatosensory dance games involve a multitask format that incorporates training of motor, coordination, rhythm, reaction, memory, executive function, and other cognitive domains. In addition, the social participation and pleasure of playing games with others also lessen the risks of loneliness, social isolation, and depressive symptoms [3].

Our observations of reduced body weight in the intervention participants were concordant with those of past studies [46]. Moreover, time spent sitting on weekdays diminished after the intervention compared with baseline levels, consistent with previous findings that somatosensory games increase physical activity [7,47]. Even with the impact of the COVID-19 pandemic, participants changed their lifestyles and daily activities through regular participation in digital somatosensory dance games, which implies potential long-term benefits of playing digital somatosensory dance games in promoting healthy aging.

Associations between higher homocysteine levels and cognitive impairment have been repeatedly demonstrated [48,49]. In our study, intervention group participants had comparatively lower serum homocysteine levels, which may be relevant to important theories of neuroinflammation and DNA methylation in the development of Alzheimer disease [50]. Nevertheless, our study showed some muscle mass loss, which is inconsistent with the reduced serum levels of homocysteine in other intervention studies [51]. Similar to other studies showing the adverse effects of the COVID-19 pandemic on health promotion intervention activities [52], the reduction in muscle mass and calories expended per day in both groups may be explained by the interruption of interventions and restricted daily activity related to pandemic control measures.

### Limitations

　　Despite its merits, this RCT had several limitations, particularly the impact of the COVID-19 pandemic, which restricted and interrupted participation in the intervention program due to disease control measures. This may have consequently attenuated beneficial effects of the intervention. In addition, the pandemic also adversely affected psychosocial well-being, which may have counteracted possible intervention effects on resilience and demoralization. Notably, quality of life was different between groups, which supports the effect of gamification. The predominance of female participants, reflecting that women tend to participate in recreational activities more than men, may also have led to sex-specific effects on the study results [3-5]. Given these limitations, the evident favorable effects of digital somatosensory dance game participation on cognitive performance and brain signatures are very encouraging for healthy aging. Although digital somatosensory dance game participation benefits older adults, it is important to generalize to availability within community settings and to further evaluate the effectiveness and sustainability of such interventions.

### Conclusions

Participation in digital somatosensory dance games for 6 months not only facilitated structural and functional alterations in various brain regions associated with somatosensory, motor, visual, and attentional functions but also improved physical and mental health. Our findings also suggest that distinctive alterations in the reward system and cerebellum play a significant role in improving motor function, skilled movements, and motivation. Gamifying interventions may provide a potentially sustainable, effective approach to promoting healthy aging and may be considered an effective component of community-based, multidomain, healthy aging programs.

## Appendix

Multimedia Appendix 1 Further details of the dance intervention.

Multimedia Appendix 2 CONSORT checklist.

## Abbreviations

3D-MPRAGE: 3-dimensional magnetization-prepared rapid-acquisition gradient-echo
AFNI: Analysis of Functional Neuroimaging
BRS: Brief Resilience Scale
DARTEL-VBM: Diffeomorphic Anatomical Registration Through Exponentiated Lie Algebra voxel-based morphometry
fALFF: fractional amplitude of low frequency fluctuations
FDR: false discovery rate
FSL: Functional Magnetic Resonance Imaging of the Brain Software Library
FWHM: full-width at half-maximum
GBC: global brain connectivity
GMV: gray matter volume
IPAQ: International Physical Activity Questionnaire
KCC: Kendall coefficient of concordance
MNI: Montreal Neurological Institute
MoCA: Montreal Cognitive Assessment
MRI: magnetic resonance imaging
RCT: randomized controlled trial
ReHo: regional homogeneity
rsfMRI: resting-state functional MRI
SPM: Statistical Parametric Mapping
TE: echo time
TI: inversion time
TR: repetition time
WHO: World Health Organization
