# Supplementary material for: Efficacy of Digital Dance on Brain Imagery, Cognition, and Health: Randomized Controlled Trial
Source: J Med Internet Res. 2024 Jul 30;26:e57694. doi: 10.2196/57694 (PMC11322681; doi:10.2196/57694)
Supplement: Multimedia Appendix 1 [file jmir_v26i1e57694_app1.docx]

| **Table S1.** Intervention schedule, activities, completion rates, and reasons for discontinuations | | | |
| --- | --- | --- | --- |
| Intervention schedule | Activity | Intervention participants completing activity (n=30) | Reason(s) for discontinuation |
| Pre-intervention | Baseline data: MRI; questionnaires (cognitive performance, physical activity levels, resilience, demoralization, quality of life); physical performance; body composition; biomarkers. | 30 (100%) |  |
| Weeks 1–6 | Level one (easy) dance game, ≥24 min twice weekly. | 30 (100%) |  |
| Weeks 7–13 | Level two (normal) dance game, ≥24 min twice weekly. | 29 (97%) | One participant went travelling. |
| Weeks 14–20 | Level three (hard) dance game, ≥24 min twice weekly. | 26 (87%) | Bad weather (heavy rain or cold) affected participants’ willingness to go out to join the program.  One participant travelled to visit relatives. |
| Weeks 21–26^a^ | Level four (master) dance game ≥24 min twice weekly. | 27 (90%) | Three participants could not achieve level 4 due to limited physical flexibility. |
| Post-intervention | Follow-up data: MRI; questionnaires; physical examination; biomarker assays. | 26 (87%) | COVID-19 pandemic limited participants’ post-test attendance; for example, some stated abroad or elsewhere in Taiwan. |
| MRI, magnetic resonance imaging.  ^a^Level 4 dance game sessions were discontinued at for 1 month due to the COVID-19 pandemic, then recommenced afterwards at level 3 or 2. | | | |

| **Table S2.** Digital dance game categories of dance complexity, difficulty levels, and scoring system | | |
| --- | --- | --- |
| Dance | Level of difficulty | Rank |
| Assigned dance 1  (One-star) | Level 1 (Easy)  30 specific marked locations | Miss |
|  |  | Nice |
|  |  | Great |
|  |  | Perfect |
|  | Level 2 (Normal)  40 specific marked locations | Miss |
|  |  | Nice |
|  |  | Great |
|  |  | Perfect |
|  | Level 3 (Hard)  52 specific marked locations | Miss |
|  |  | Nice |
|  |  | Great |
|  |  | Perfect |
|  | Level 4 (Master)  52 specific marked locations | Miss |
|  |  | Nice |
|  |  | Great |
|  |  | Perfect |
| Assigned dance 2  (Two-star) | Level 1 (Easy)  64 specific marked locations | Miss |
|  |  | Nice |
|  |  | Great |
|  |  | Perfect |
|  | Level 2 (Normal)  92 specific marked locations | Miss |
|  |  | Nice |
|  |  | Great |
|  |  | Perfect |
|  | Level 3 (Hard)  162 specific marked locations | Miss |
|  |  | Nice |
|  |  | Great |
|  |  | Perfect |
|  | Level 4 (Master)  162 specific marked locations | Miss |
|  |  | Nice |
|  |  | Great |
|  |  | Perfect |
| Assigned dance 3  (Three-star) | Level 1 (Easy)  57 specific marked locations | Miss |
|  |  | Nice |
|  |  | Great |
|  |  | Perfect |
|  | Level 2 (Normal)  109 specific marked locations | Miss |
|  |  | Nice |
|  |  | Great |
|  |  | Perfect |
|  | Level 3 (Hard)  173 specific marked locations | Miss |
|  |  | Nice |
|  |  | Great |
|  |  | Perfect |
|  | Level 4 (Master)  173 specific marked locations | Miss |
|  |  | Nice |
|  |  | Great |
|  |  | Perfect |

**Table 3.** Digital dance game score determination algorithm

0 seconds

Start song

t seconds

Activate scoring system

t + p seconds

Determine: Perfect

Determine: Great

t + g seconds

Determine: Nice

t + n seconds

Determine: Miss

Determining criteria: 1. Scope of time frame: t < t + p < t + g < t + n

2. Scope of radius: Rp ≤ Rg ≤ Rn

**Figure S1.** Digital dance game, showing criteria determining score ranking


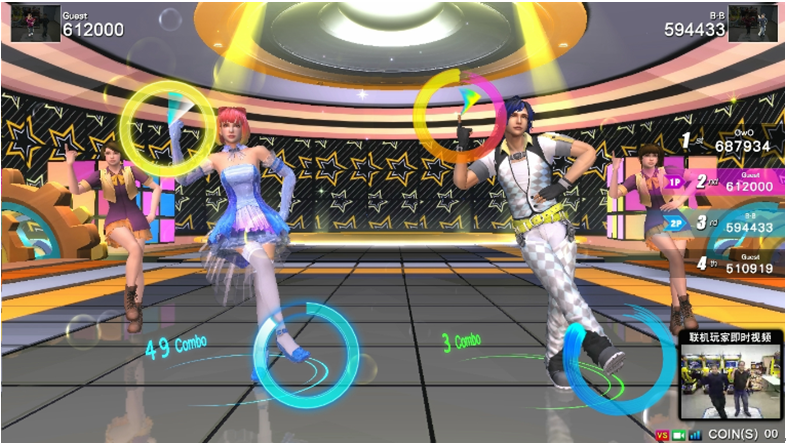


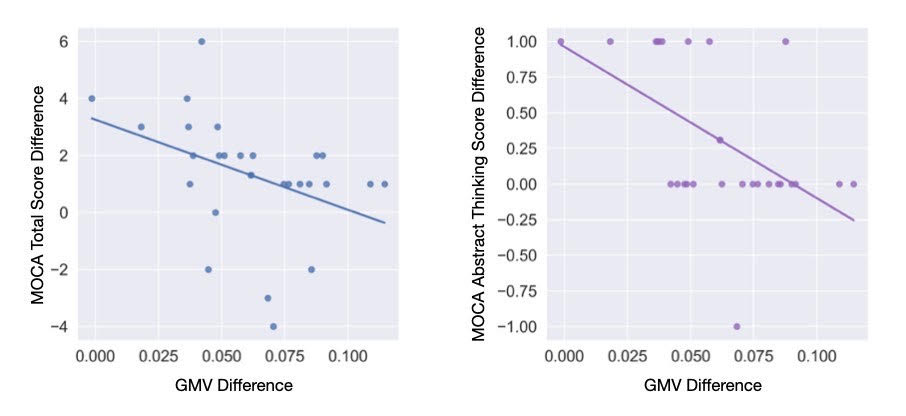


**Figure S2.** Association of MOCA total score/ sub-domain abstract thinking with brain imagery changes of GMV
